# Characterizing Macrophages Diversity in COVID-19 Patients Using Deep Learning

**DOI:** 10.3390/genes13122264

**Published:** 2022-12-01

**Authors:** Mario A. Flores, Karla Paniagua, Wenjian Huang, Ricardo Ramirez, Leonardo Falcon, Andy Liu, Yidong Chen, Yufei Huang, Yufang Jin

**Affiliations:** 1 Department of Electrical and Computer Engineering, University of Texas at San Antonio, San Antonio, TX 78249, USA; 2 Department of Electrical Engineering and Cyber Engineering, Houston Baptist University, Houston, TX 77074, USA; 3 McKetta Department of Chemical Engineering, University of Texas at Austin, Austin, TX 78712, USA; 4 Greehey Children’s Cancer Research Institute, University of Texas Health San Antonio, San Antonio, TX 78229, USA; 5 Department of Population Health Sciences, University of Texas Health San Antonio, San Antonio, TX 78229, USA; 6 Department of Medicine, School of Medicine, University of Pittsburgh, Pittsburgh, PA 15232, USA; 7 UPMC Hillman Cancer Center, University of Pittsburgh, Pittsburgh, PA 15232, USA

**Keywords:** deep learning, single-cell RNA-Seq, SARS-CoV-2, cell type identification, infection severity

## Abstract

The severe acute respiratory syndrome coronavirus 2 (SARS-CoV-2), the etiological agent responsible for coronavirus disease 2019 (COVID-19), has affected the lives of billions and killed millions of infected people. This virus has been demonstrated to have different outcomes among individuals, with some of them presenting a mild infection, while others present severe symptoms or even death. The identification of the molecular states related to the severity of a COVID-19 infection has become of the utmost importance to understanding the differences in critical immune response. In this study, we computationally processed a set of publicly available single-cell RNA-Seq (scRNA-Seq) data of 12 Bronchoalveolar Lavage Fluid (BALF) samples diagnosed as having a mild, severe, or no infection, and generated a high-quality dataset that consists of 63,734 cells, each with 23,916 genes. We extended the cell-type and sub-type composition identification and our analysis showed significant differences in cell-type composition in mild and severe groups compared to the normal. Importantly, inflammatory responses were dramatically elevated in the severe group, which was evidenced by the significant increase in macrophages, from 10.56% in the normal group to 20.97% in the mild group and 34.15% in the severe group. As an indicator of immune defense, populations of T cells accounted for 24.76% in the mild group and decreased to 7.35% in the severe group. To verify these findings, we developed several artificial neural networks (ANNs) and graph convolutional neural network (GCNN) models. We showed that the GCNN models reach a prediction accuracy of the infection of 91.16% using data from subtypes of macrophages. Overall, our study indicates significant differences in the gene expression profiles of inflammatory response and immune cells of severely infected patients.

## 1. Introduction

The current global pandemic situation of COVID-19 due to the SARS-CoV-2 virus has affected the lives of billions. As a highly transmissible and pathogenic coronavirus that emerged in late 2019 and has caused a pandemic of acute respiratory disease [1], the SARS-CoV-2 virus is related to the original SARS-CoV, which was highly lethal but faded out after intense public health mitigation measures [2]. One of the mysteries of COVID-19 is why some people suffer severe symptoms, even life-threatening complications, while others suffer no symptoms or just mild ones.

Several studies have related the severity of a COVID-19 infection to immune system features resulting in groups more vulnerable to this viral infection [3,4]. Further, recent studies have illustrated the special roles of macrophages and monocytes in the inflammatory response to COVID-19 [5,6]. It has also been shown that in severe cases of COVID-19, the virus promotes a cytokine storm with an uncontrolled massive release of pro-inflammatory cytokines, leading to acute respiratory distress syndrome (ARDS) and acute heart failure, and these conditions are highly life-threatening and fraught with the acquisition of secondary bacterial infections [7]. The quantitative profiles of the immune cell subsets and molecular factors associated with protective or pathological immunity against severe COVID-19 can potentially help in gaining a molecular understanding of this pandemic disease and in the development of vaccines and therapeutics [8,9,10,11]. However, the characterization of cell types related to COVID-19 infection severity has not been well defined.

The characterization of subtypes of macrophages in patients with different levels of COVID-19 severity can be accomplished using single-cell technologies. In particular, scRNA-Seq has become mature enough to provide answers to complex research questions found in the study of dysregulation of the immune systems observed in COVID-19 patients. Several studies on COVID-19 infection using scRNA-Seq technology have been reported recently [8,9,10,11], paving a foundation to explore different cell types involved in the COVID-19 infection severity.

The use of single-cell profiling led to a significant increase in the amount of data collected, which results in computational challenges in processing massive and complicated datasets. To address these challenges, deep learning (DL) is positioned as a competitive alternative for single-cell analyses besides the traditional machine learning approaches [12]. In this work, we applied two major computational analyses. First, we implemented a customized single-cell analysis pipeline that included normalization, batch correction, integration, dimensionality reduction, and cell-type prediction to determine the cellular profiles in healthy controls and patients with different severity of COVID-19 symptoms. We then developed deep learning models to predict COVID-19 severity using gene expression profiles of cells in a specific cell type, macrophages. The results of this work show significantly different cell compositions in mild (7316 cells) and severe (37,197 cells) groups compared to normal (19,221 cells). Importantly, inflammatory responses were dramatically elevated in the group with severe symptoms, as well as decreased populations of T cells.

## 2. Materials and Methods

scRNA-Seq datasets with thirteen patients were downloaded from NCBI GEO under the accession number GSE145926 [8]. A total of 12 BALF samples that included six patients with severe symptoms (S), three patients with mild symptoms (M), and three healthy control patients (N) were analyzed. In the original dataset, healthy control, patients with mild symptoms, and those with severe symptoms were denoted as HC, O, and S/C, respectively. We excluded from our study one healthy control sample whose genomic data were originally collected in a different study and lacked as detailed patient information as the other 12.

Data were pre-filtered to remove doublets or potential dead cells under the following criteria. (1) The number of genes detected in a cell is between 200 and 6000. (2) The Unique Molecular Identifier (UMI) counts in a cell should be greater than 1000. (3) The mitochondrial (MT) percentage is smaller than 10%. A total of 23,916 genes and 63,734 cells were obtained after the filtering process.

### 2.1. Normalization and Batch Effect Correction

Cell-to-cell normalization of each patient by negative binomial regression was performed using scTransform from the Seurat v3 package in R [13,14,15]. The 12 samples (patients) were clinically pre-classified into three groups, N, M, and S. Batch effect correction was then performed for samples in each group to eliminate the technical noise, remove variations between cells, and align samples in the same group while avoiding removing biologically relevant data. The “anchor” method from Seurat v3 was used for the batch effect correction [13].

### 2.2. Dimension Reduction and Clustering

Principal Component Analysis (PCA) was performed for dimensionality reduction over the first 30 components using Seurat. A total of 2000 highly variable features (genes) were selected. Clusters were identified using the Leiden algorithm with a resolution of 1.21 from Seurat v3 [16].

### 2.3. Cell Type Identification

Cell types were assigned to each of the clusters obtained from 2.2 using the automated method from Ding et al. [17]. We calculated the affinity of a cell to a candidate cell type using the marker genes from the CellMatch and CellMarker databases of marker genes [18] as follows:

(1) $$s_{c}^{t}=\log\left(\frac{\sum_{c}^{M_{t}}x_{y,c}}{K_{c}}\;10^{4}+1\right),$$

where $s_{c}^{t}$ represents the score of a cell c belonging to a candidate cell type t. $M_{t}$ represents marker genes for each candidate cell type t. The variable $x_{y,c}$ represents the UMI count of the marker gene y in a cell c. The variable $K_{c}$ denotes the total UMI count in cell c. By calculating $\frac{\sum_{c}^{M_{t}}x_{y,c}}{K_{c}},\;$ the contribution of marker genes’ expressions to the overall gene expression in a cell c for a cell type *t* is evaluated. Scale factors, 10^4^ and 1, are introduced to facilitate the logarithm calculation.

For a cluster of cells, the scores for each cell belonging to a cell type *t* can be obtained using Equation (1). For a given cluster and a given cell type *t*, a cell in that cluster is a true positive if the score $s_{c}^{t}$ is above a given threshold and a false negative otherwise. On the other hand, a cell not in that cluster is a false positive if it has a score above the threshold and a true negative otherwise. A receiver operating characteristic curve was plotted to show the true positive rate against the false positive rate at different thresholds. The Aera Under Curve (AUC) is 1.0 for perfectly assigning a cell type to a cluster, and around 0.5 for randomly assigning a cell type to a cluster. Specifically, for each cluster, the cell type with the highest AUC was assigned to that cluster if the highest AUC score for a cell type is 5% larger than the second highest AUC score. If AUC scores for different cell types are similar, a cell type with a larger number of gene markers enriched and a higher percentage of cells expressing the marker genes will be considered.

### 2.4. Deep Learning Models to Predict COVID-19 Infection Severity with Gene Expressions

Two different DL models, Artificial Neural Network and Graph Convolutional Neural Network, have been deployed to predict COVID-19 infection severity using gene expressions in assigned cell types.

We adopted the ANN model since it is the easiest fully connected model to implement. GCNN models were also established by integrating biological gene–gene interactions into the models, hoping for a better performance of the models. In this study, a GCNN model includes an input graph represented by an adjacency matrix, graph convolutional layers (coarsening and pooling), and a hidden layer connected to an output layer with three nodes representing the N, M, and S groups.

The database-driven network graph is taken from the GeneMania database (https://GeneMania.org/ accessed on 1 September 2021) for the GCNN models [19]. GeneMania has a large number of interactions and incorporates both gene-to-gene and protein-to-protein interactions. Since the input gene expression profiles for all models are consistent, the GeneMania graph does not change and is established once for all models. A *p*-value threshold was also established for the GeneMania graph to keep only interactions with the confidence of (*p* < 3 × 10^−5^) from the filtered genes obtained after dimensionality reduction in Section 2.2. This threshold was chosen to obtain a sufficient number of connections while minimizing the number of singleton nodes. We have applied both ANN and GCNN models in other studies and the graph convolution algorithms and codes for GCNN are available at Github (https://github.com/Karladanielap/GeneSignaturesCOVID19 accessed on 1 September 2021). The convolution algorithm of the GCNN layer can be found in our previous research [20,21].

**Figure 1:**
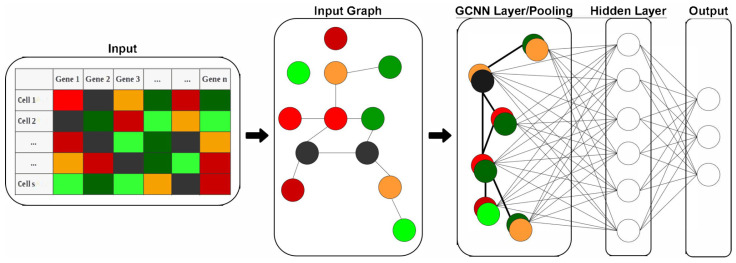
Structure of the developed GCNN model. The model includes a graph convolution layer and a fully connected output layer for classification. Inputs to the GCNN models are expression levels of 2000 genes in each cell and an input graph. The input graph includes 2000 genes as nodes, and edges among nodes representing gene-to-gene interactions from the GeneMania database. The input graph is then pooled into a single GCNN layer which will be fed into the hidden and output layers.

We trained two sets of models. The first set of models includes both ANN and GCNN models using gene expressions from a cell as input, while the output includes COVID-19 infection severity levels. We did not integrate cell-type information in this set of models. The second set of models only uses gene expression profiles from M1, M2, and macrophages identified in our study to predict the COVID-19 infection severity as the output. We termed this a macrophage-specific (M-specific) ANN model or a macrophage-specific GCNN model, as shown in Figure 1.

### 2.5. Differential Gene Expression and Gene Ontology Analysis

Differential gene expression analysis was performed in R through a widely adopted package, Model-based Analysis of Single-cell Transcriptomics (MAST) [22]. We further filtered the genes considered differentially expressed with a threshold of *p*-value < 0.05 and fold change (FC) > 2 (up-regulated) or FC < 0.5 (down-regulated) to keep only the significantly differentially expressed genes (DEG) [23]. We analyzed the obtained genes with Database for Annotation, Visualization, and Integrated Discovery (DAVID) [24], a program that integrates functional and genomic annotations with intuitive graphical summaries, to obtain the gene ontology (GO) terms of the significantly differentially expressed genes.

## 3. Results

### 3.1. Integration of scRNA-Seq Data of COVID-19 Patients Classified by Severity Produces a High-Quality Normalized Dataset

To understand the cell type and molecular differences for COVID-19 patients with different degrees of severity, we implemented a custom pipeline to re-process public scRNA-Seq datasets. After pre-processing, we obtained 63,734 cells with 23,916 genes for analysis. The distribution of the number of genes detected, the UMI, and the percentage of MT in cells are illustrated in Figure S1. The violin plots showed that most cells harbor a 2% MT content, less than 10,000 UMIs, and less than 2000 genes, suggesting high-quality cells supported by a good number of UMI reads. Demographics of each patient in the study, together with the number of cells detected for each patient, were also included (Table 1). We observed high variations in the number of cells for each sample, reflecting variations in the quality of the samples that were corrected during the normalization and integration steps of our customized scRNA-Seq pipeline.

### 3.2. Differences in the Number of Clusters across Conditions Suggest a Correlation to COVID-19 Infection Severity

To evaluate if there were differences in the number of clusters across conditions, we processed samples from each condition using PCA. The top 2000 highly variable genes were selected from the original 23,916 genes and the resulting data were processed. To further verify how the changes in clusters relate to COVID-19 infection severity, we performed batch effect correction and integration for all samples (12 patients).

**Figure 2:**
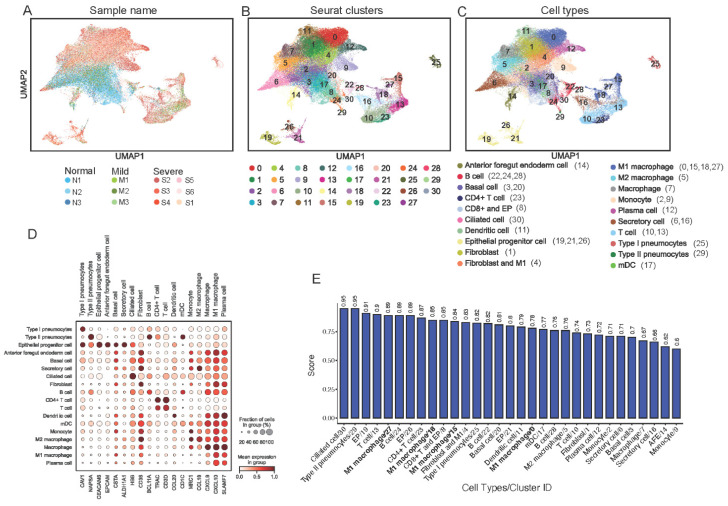
Alignment and clustering of gene expression from 12 patients after normalization and batch effect correction. (**A**) Cells from all 12 patients in N, M, and S groups were visualized using UMAP; (**B**) A total of 31 clusters were identified for potential cell-type assignments; (**C**) Cell types assigned to each cluster were visualized using UMAP. Cell type names and their corresponding cluster numbers (in parenthesis) were provided in legend; (**D**) Dot-plot of cell types assigned with selected gene markers. Each gene marker listed at the bottom is a selected marker for a specific cell type listed on top of the subfigure. (**E**) The AUC scores for cell type assignment to each cluster illustrated the confidence of cell type identification.

We observed that, in each group, cells were distributed uniformly, suggesting the good performance of normalization and batch effect correction for the three groups (see Figure 2A). We found a total of 31 clusters assigned to 20 cell types (Figure 2B,C). In Figure 2D, a larger and darker dot represented the percentage of cells in a cluster expressed in the selected marker gene (expression level > 0). We noted that cell types should be determined by multiple markers and a full list of the gene markers used is shown in Table S1. The gene marker *NAPSA* represents type II pneumocytes with a darker and larger dot compared with *NAPSA* expressions in other cell types in Figure 2D; correspondingly, the AUC score for type II pneumocytes is very high (AUC = 0.95), suggesting a higher confidence to assign type II pneumocytes to cluster 29. A cell type might be assigned to multiple clusters; for example, M1 macrophages were assigned to clusters 0, 15, 18, and 27 with different AUC scores (Figure 2E). As a marker gene for macrophages, *CXCL10* was also highly expressed in other cell types; however, *CXCL10* should not affect other cell type assignments if it was not one of the marker genes for the cell type under consideration.

The number of cells and percentage of cells in each cluster for 12 patients were shown in Table 2. A total of 20 clusters of cells were found across all 12 patients. We also found differences in the percentage of cells from each condition (normal, mild, and severe) that composed the cluster, suggesting that these differences might be related to COVID-19 infection severity.

### 3.3. Clustering with Respect to COVID-19 Severity Levels Suggests Disease-Related Cell Activation

**Figure 3:**
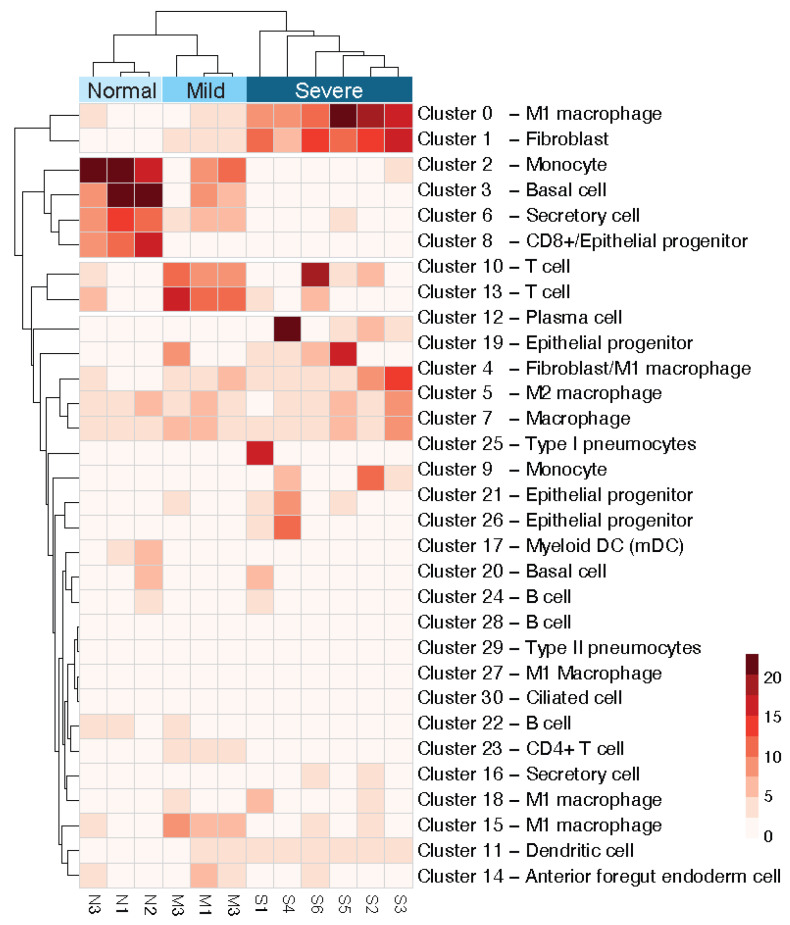
Clustergram of the percentage of cells in each cluster from each patient and their COVID-19 infection severity. The *X*-axis displays the patients where normal, mild, and severe symptoms patients are clustered together. The *Y*-axis shows the 31 clusters numbered from 0 to 30 and the corresponding cell types assigned. Clusters get darker red in the heatmap where there is a higher percentage of cells that belong to a specific patient.

To further test if clusters of cells are related to severity levels, we calculated the percentage of clusters in each patient shown in Table 2. We applied a hierarchical clustering algorithm based on the percentage of cells in a cluster for a patient and the attribute of a patient’s group (N, M, S) (Figure 3). The profiles of cell clusters successfully classified the N, M, and S groups, suggesting cellular activation profiles of patients representing the severity of COVID-19 infection.

### 3.4. Cell Type Assignment

Liao et al.’s paper presented an excellent preliminary study to identify 11 cell types (Ciliated, Secretory, Macrophages, Neutrophil, mDC, pDC, Mast cell, T cell, NK, B cell, and Plasma cell) and others with a total of 12 gene markers listed (*TPPP3*, *KRT18*, *CD68*, *FCGR3B*, *CD1C*, *CLEC9A*, *LILRA4*, *TPSB2*, *CD3D*, *KLRD1*, *MS4A1*, *IGHG4*), as shown in Extended Data Figure 1 [8]. Having noticed that two major cell types, alveoli, and fibroblasts, were not reported, and that one cluster in Liao et al.’s study was not assigned to any cell type (denoted as others), it motivated us to perform cell-type identification, aiming for a more complete catalog of cell types and subtypes.

Our study has assigned all 63,734 cells to one of the 20 cell types (see Table 3). Specifically, we identified more subtypes of cells; for example, Liao et al.’s results only showed macrophage clusters, while we have identified three subtypes, including macrophages, M1 macrophages, and M2 macrophages (87.1% overlapping with Liao’s macrophage cell type). Identification of subtypes of macrophages is important since M1 macrophages and M2 macrophages have different regulatory roles in inflammatory responses. Moreover, 98.8% of the epithelial subtypes, including secretory, ciliated, basal, or epithelial progenitor (EP) cells, that we identified were identified only as epithelial cells. About 68% of the cells we identified as subtypes of T cells (T cell and CD4+ T cell) were previously identified only as T cells.

To confirm our cell type assignment, we also compared the cell composition of the normal group with other studies and our results agreed with the reported cell compositions [25,26,27,28,29,30]. In Table 3, about 20.97% (5.67% macrophages, 5.39% M2 macrophages, and 9.91% M1 macrophages) and 34.11% (6.01% macrophages, 5.79% M2 macrophages, and 22.31% M1 macrophages) of cells identified were macrophages from samples with mild and severe symptoms, respectively, suggesting elevated inflammatory responses in mild and severe groups. Fibroblasts accounted for 0.73%, 4.13%, and 14.35% of cells identified from samples as normal, mild, and severe symptoms, respectively, indicating possible structural changes in the infected lungs. Interestingly, it is reported that the proportions of macrophages significantly increased from 12% in normal (with single-nucleus RNA seq data) to 20% in lung tissue with COVID-19 infection, as well as those of fibroblasts, from 7% in normal to 23% in infected lungs [31]. The identified sub-types of cells in our study provide a more detailed picture of the cell-type composition and its dysregulation related to COVID-19 infection severity.

**Figure 4:**
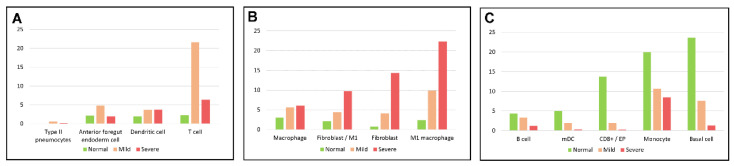
Trends of cell population changes in 13 cell types shared in the normal, mild, and severe groups with the *Y*-axis representing the percentage of cells in N, M, and S. (**A**) The proportion of cell types demonstrated a “Λ” shape in the normal, mild, and severe groups. (**B**) The proportion of cell types increased in the mild and severe groups. (**C**) The proportion of cell types decreased in the mild and severe groups.

Table 3 lists the percentage of cell types in each group where we can observe trends of populations of 13 cells types, including Type II pneumocytes, anterior foregut endoderm (AFE) cell, T cell, dendritic cell, Macrophage, fibroblast/M1 macrophage, fibroblast, M1 macrophage, B cell, mDC, CD8+/EP, monocytes, and basal cells, identified in N, M, and S groups (See Figure 4). T cells and AFE cells demonstrated a “Λ” shape, with an increased percentage in the mild group and a decreased percentage in the severe group compared to the normal group (Figure 4A). As an indicator of immune defense, populations of T cells (T cells and CD4+ T cells) accounted for 24.76% in the mild group and decreased to 7.35% in the severe group. Cell proportions of monocytes, basal cells, and mDC in the mild and severe groups significantly decreased compared to the normal group (Figure 4C). Since macrophages were differentiated from monocytes, decreased monocyte populations (Figure 4C) were observed with the increased populations of macrophages (Figure 4B).

### 3.5. Deep Learning Models for COVID-19 Infection Severity Prediction Supported by Significant Differences in the Gene Expression Profiles of Subtypes of Immune Cells

One ANN model and one GCNN model were trained with 80% of cells from each patient without consideration of cell types, while M-specific ANN and M-specific GCNN models were trained with cells identified as M1, M2, and macrophage in Section 3.4.

ANN models include one input layer with 2000 nodes for gene expressions, one hidden layer with 32 nodes, and one output layer with three nodes representing the normal, mild, and severe levels of infection. The model was trained with the dropout rate as 0.5, the learning rate as 0.0006, the batch size as 128, softmax activation, adam optimizer, and sparse–categorical cross–entropy loss function using the Keras package.

GCNN models were also developed for all cell types and macrophages specifically with the model structure. The GCNN models include one graph with 2000 nodes (genes) and 199,900 edges. After the input graph, a hidden layer with 128 nodes with softmax activation was introduced, as well as a fully connected output layer with three nodes representing the normal, mild, and severe infection levels. The parameters for GCNN models were chosen as follows: dropout rate is 0.5, the learning rate is 0.0005, and the batch size is 128.

A total of 15 data partitions were established with 80%, 10%, and 10% of cell types extracted from each patient to train, validate, and test the models. Both ANN and GCNN models were developed, trained, and tested with the same data partitions. All ANN and GCNN models have comparable training losses. Average and the best performances of ANN and GCNN models with 15 partitions were presented in Table 4. The best performance of the M-specific GCNN model has a testing performance of 91.48% and beats all other models. Prediction accuracy generated from an M-specific GCNN model was shown in Table 5. The prediction accuracy for the mild group was the worst due to a smaller sample size compared with severe and normal groups, as shown in Table 1.

We also performed classification using logistic regression in scikit-learn by using a 5-fold cross-validated grid parameter search. The best parameters across all searched parameters are inverse regularization of 10 and L_1_ penalty using linear optimizer. The logistic regression model has achieved the best score of 82.9%, and the testing set has the best score of 82.9%. The average prediction accuracy of ANN and GCNN modes using all cell types reached a performance similar to the logistic regression approach and did not show significant improvement. However, gene expression profiles in identified macrophages significantly improved the prediction accuracy, suggesting that the characterization of macrophages might be related to COVID-19 infection severity.

### 3.6. A Subtype of M1 Macrophages Is Associated with Severe COVID-19 Cases

With the cell type identified, we further examined if cells in a cluster are observed in samples from a unique group (See Table 6). In cluster 0, which is assigned as M1 macrophage, 93.2% of cells belong to severe samples, 3.45% to mild samples, and 3.35% to normal samples (see Table 6). Similarly, cells from clusters 5, 7, 15, and 18 belong to samples from the N, M, and S groups. Cells from cluster 27 (M1 macrophages) are only observed in samples from the M and S groups.

Since M1 macrophages were assigned to multiple clusters, we further examined the DEG in these clusters to see if we can assign a subtype to a cluster. A total of 142 genes were found to be differentially expressed (90 for up-regulated, and 52 for down-regulated) in cluster 0 (See Tables S4 and S5) compared to the other M1 macrophage clusters (15, 18 and 27). Interestingly, all the 90 up-regulated DEGs and 51 down-regulated DEGs were only found in cluster 0 and not in the other clusters (see Table 7 for unique DEGs). The number of DEGs and unique DEGs in cluster 0 are significantly higher than the other clusters designated as M1 macrophages, suggesting a possible subtype of M1 macrophages.

To further investigate if this is a subtype of M1 macrophages, we examined the biological processes enriched by the DEG with larger FC (see Table S2 for up-regulated genes and S4 for down-regulated genes). The enriched biological processes of up-regulated genes include chemotaxis, cytokines, immunity, inflammatory response, antiviral defense, and apoptosis (Table S3), which are strongly associated with the COVID-19 infection. The DEGs with larger FC include the alarmins *S100A8*, *S100A9*, *CXCL10* and *CCL2*. *S100A8* and *S100A9* are endogenous molecules released in response to environmental triggers and cellular damage. They are constitutively expressed in immune cells and their expression is up-regulated under inflammatory conditions [32] (see Table 7). *CXCL10* and *CCL2* have been reported as key players in the onset and maintenance of cytokine storm in severe cases of COVID-19 [33]. Additionally, the expression levels of *GNLY*, *GSZMB*, and *CCL5* were significantly downregulated (see Table 8). It is well-known that *GNLY* functions as an antimicrobial peptide [34]. *GSZMB* deficiency exacerbates lung inflammation in mice after acute lung injury [35]. Moreover, low levels of *CCL5* have been associated with severe COVID-19 infection [36]. Based on these results, we speculate that this subtype of M1 macrophages in cluster 0 is an intermediate subtype related to immunity, inflammatory responses, and cytokine storm in COVID-19 infection.

In particular, we identified two genes (*APOBEC3A* and *IDO1*) that are unique to this cluster and that may also be considered as gene markers for the M1 subtype. *APOBEC3A* has a key role in cytidine deaminase for transcriptomic and functional polarization of M1 macrophages [37], while *IDO1* plays a potential role in macrophage differentiation where the expression levels of this gene modulate macrophage differentiation. Previous findings support the role of *IDO1* with regard to the polarization of macrophages to restrain excessive or inappropriate immune activation in inflammatory or tumor microenvironments [38].

## 4. Discussion

This is the first study to line up the characterization of macrophages to the severity of COVID-19 infections using a single-cell RNA Seq analysis. A total of 31 cell clusters were found in a previously published dataset and the percentages of the cell clusters from 12 samples were used to successfully predict the severity of the COVID-19 infections. To gain a better understanding of the specific cellular responses to COVID-19 infections, these 31 cell clusters were further mapped into 20 cell types with well-defined gene markers in the lungs. Trends of the cell profiles in the normal, mild, and severe groups were then compared. The most significant changes for the immune system and inflammatory responses were found in macrophages, monocytes, and T cells, while for lung function and structures, they were found in fibroblasts, EP, and basal cells. Different cell proportions identified in the normal, mild, and severe groups triggered a further characterization of specific cell types. ANN and GCNN models were developed to predict COVID-19 infection severity with gene expressions in all cell types and with gene expressions from M1+M2+macrophages, considering that macrophages are the most significant cell type changes among the normal, mild, and severe groups. Our results showed that the macrophage-specific GCNN model had the highest prediction accuracy, confirming the significant role of macrophages in predicting the severity of COVID-19.

The novelty of this study lies in the integration of a single-cell RNA seq analysis with DL models to predict the severity of COVID-19 infection. Due to the complexity of single-cell data, a significant research effort was allocated at the early stage for normalization and batch effect correction to reduce technical experimental variations and individual differences among batches and cells, while keeping meaningful biological information. Though multiple scRNA-Seq pipelines such as Seurat [13] and Scanpy [39] are available for users to perform the analysis, the rationale for selecting special thresholds during the analysis should be carefully examined by users with a good understanding of both statistical analysis and biological processes. Overwhelming batch correction may lead to the loss of biological information for a scRNA-Seq analysis.

With the availability of DL modeling tools such as Keras, training DL models is getting easier, while the interpretation of DL remains premature. We adopted a feature selection-searching approach in this study. The completeness and soundness of the model should be further investigated in future studies. There are other approaches to establishing a GCNN graph for scRNA-Seq data, including using a cell–cell graph or a gene-to-cell graph [40,41]. Since the goal of this study was to line up gene expression profiles to cell types and then infection severity, we first established a data-driven graph from genes to cell clusters, borrowing the idea reported in [41]. However, the performance of the GCNN with gene-to-cell cluster graph was not as good as the GCNN models presented here. We examined the edges in the data-driven gene-to-cell cluster graph and found the number of edges was much smaller than that in other graphs used before. In addition, since there is no backward prorogation to refine the weights of the edges in the gene-to-cell cluster graph, any error introduced in the graph will stay there and affect the prediction accuracy. On the other hand, the adopted gene-to-gene interaction graph is a pure knowledge-driven graph. Thus, the errors introduced to the graph are controllable based on prior knowledge. One possible way to improve the performance of the GCNN model is to establish a pure biology-driven graph combining gene–gene interactions and links from marker genes to specific cells for GCNN models in the future.

Our study indicates significant differences in the gene expression profiles of subtypes of immune cells of COVID-19-infected patients. The molecular components of these profiles deserve further research and experimentation as potential therapeutical factors.

## Figures and Tables

**Table 1:** Demographics of patients and number of cells after filtering. The average age of 12 patients is 45.91 with a standard deviation of 16.1.

Sample	Number of Cells	Gender	Age	Chronic Disease
Normal1	8466	Female	38	-
Normal2	8189	Male	24	-
Normal3	2566	Male	22	-
Mild1	3542	Male	36	-
Mild2	3411	Female	37	-
Mild3	363	Male	35	-
Severe1	17,340	Male	62	-
Severe2	1292	Male	66	Hypertension
Severe3	1718	Male	63	Sleep apnea
Severe4	2071	Female	65	Diabetes
Severe5	2904	Female	57	-
Severe6	11,872	Male	46	-

**Table 2:** The percentage of cells in each cluster for each patient. N, M, and S represent severity groups, respectively, while the number after the group represents the patient ID in the corresponding group; for example, N1 represents patient 1 in the normal group. Note: the summation of percentage in each column is 1, representing the cell composition of a sample (see also Figure S2).

Cluster #	Cell Type	N1	N2	N3	M1	M2	M3	S1	S2	S3	S4	S5	S6
Cluster 0	M1 macrophage	0.93	0.53	3.62	2.82	3.34	2.48	18.82	15.46	10.06	23.11	12.75	8.4
Cluster 1	Fibroblast	0.85	0.31	1.68	4.43	3.96	2.75	14.12	17.17	6.81	10.48	12.94	10.78
Cluster 2	Monocyte	21.95	15.59	20.81	9.01	11.96	1.65	1.26	3.38	1.47	1.34	1.06	0.55
Cluster 3	Basal cell	20.67	22.65	8.85	7.99	6.65	2.48	0.27	1.26	0.54	0.7	0.34	0.72
Cluster 4	Fibroblast and M1	2.45	1.48	3.27	3.42	5.66	2.75	9.36	14.21	3.48	3.84	4.49	3.89
Cluster 5	M2 macrophage	4.93	5.59	4.68	5.79	5.13	3.86	4.5	8.8	4.72	7.57	4.73	1.41
Cluster 6	Secretory cell	13.13	10.58	9.9	5.31	7.15	3.86	2.06	1.53	1.55	2.79	0.43	2.41
Cluster 7	Macrophage	2.98	2.87	3.51	6.15	5.04	6.89	4.77	9.11	5.11	5.3	4.54	3.1
Cluster 8	CD8+ T and EP	12.6	16.83	7.83	2.17	1.64	2.2	0.21	0.14	0.7	0.41	0.53	0.62
Cluster 9	Monocyte	0.87	0.59	1.68	0.65	0.73	1.1	11.16	2.8	6.35	1.98	2.17	1.03
Cluster 10	T cell	0.83	0.05	4.44	8.78	9.85	11.57	5.18	2.02	0.62	2.85	19.17	2.48
Cluster 11	Dendritic cell	2.02	1.67	2.22	4.29	3.11	2.48	3.44	3.93	3.79	3.55	4.25	4.2
Cluster 12	Plasma cell	0.01	0	0	0.08	0.21	0.28	5.31	5.03	22.52	3.67	2.12	0.96
Cluster 13	T cell	0.64	0.04	7.17	12.14	11.81	16.53	0.96	1.77	2.32	2.56	5.6	4.68
Cluster 14	Anterior Foregut Endoderm cell	2.16	1.58	4.09	6.04	3.84	1.65	2.01	1.68	2.32	0.52	3.38	2.31
Cluster 15	M1 macrophage	0.08	0.01	2.96	5.14	5.39	9.09	3.04	2.11	0.54	1.4	4.73	1.62
Cluster 16	Secretory cell	1.1	0.21	0.58	1.44	1.93	1.93	4.09	1.43	0.15	0.17	4.14	2.17
Cluster 17	mDC	4.24	6.75	2.14	2.29	1.67	1.1	0.18	0.66	0.23	0.23	0	0.1
Cluster 18	M1 macrophage	1.3	0.39	0.62	1.19	1.17	3.86	3.35	1.22	0.54	1.4	0.72	6.61
Cluster 19	Epithelial progenitor cell	0.04	0.11	0.09	0.99	0.94	8.54	0.92	1.36	3.87	16.88	6.42	4.06
Cluster 20	Basal cell	1.67	6.97	0.27	0.42	0.47	0.55	0.14	0.19	0.08	0.06	0.1	6.23
Cluster 21	Epithelial progenitor cell	0.21	0.39	2.38	1.55	2.05	3.03	0.87	1.95	9.67	4.95	1.93	2.58
Cluster 22	B cell	2.59	0.48	3.59	2.48	1.58	3.5	0.4	0.89	0.08	0.81	0.58	0.24
Cluster 23	CD4+ T cell	0.38	0.02	1.75	3.16	3.14	3.03	0.97	0.73	1.39	1.8	1.83	0.96
Cluster 24	B cell	1.29	4.25	0.47	0.48	0.38	0.28	0.17	0.11	0.08	0.06	0.92	3.93
Cluster 25	Type I pneumocytes	0	0	0.04	0.14	0.03	0.28	0.88	0.19	0.23	0.64	0	15.63
Cluster 26	Epithelial progenitor cell	0	0	0	0.03	0	0	0.39	0.08	10.53	0.47	0.63	4.72
Cluster 27	M1 macrophage	0	0	0	0.17	0.03	0	0.94	0.22	0.08	0.17	0	1.89
Cluster 28	B cell	0.04	0.07	0.23	0.9	0.64	0.83	0.18	0.31	0	0.06	0.05	0
Cluster 29	Type II pneumocytes	0.06	0	0.31	0.54	0.47	1.38	0.05	0.23	0.15	0.23	0.43	0.14
Cluster 30	Ciliated cell	0	0	0	0	0	0	0	0	0	0	0	1.55

**Table 3:** Percentage of a cell type assigned to cells in N, M, and S groups.

Cellular Function	Cell Type	Normal (%)	Mild	Severe
			(%)	(%)
Lung structure	Type II pneumocytes	0.068	0.55	0.15
	Type I pneumocytes	0.0052	0.1	1.73
	Secretory cell	12.26	7.79	4.57
	Basal cell	23.68	7.55	1.28
	Anterior foregut endoderm cell	2.17	4.8	1.95
	Epithelial progenitor cell	0.76	3.2	5.34
	Fibroblast	0.73	4.13	14.35
	Ciliated cell	0	0	0.12
Inflammatory	Macrophage	3	5.67	6.05
	M2 macrophage	5.18	5.39	5.79
	M1 macrophage	2.38	9.91	22.31
Immune	Monocyte	19.94	10.72	8.49
	mDC	5.03	1.94	0.32
	B cell	4.34	3.32	1.23
	T cell	2.23	21.62	6.36
	Dendritic cell	1.9	3.65	3.72
	CD4+ T cell	0.41	3.14	0.99
Blood	Plasma cell	0.0052	0.15	5.22
Undetermined	Fibroblast/M1	2.14	4.43	9.75
	CD8+/EP	13.77	1.93	0.26

**Table 4:** Performance of ANN and GCNN models using all cell types and macrophage-specific ANN and GCNN models for COVID-19 infection severity prediction.

	Average Performance 15 Partitions			Best Performance		
	Train	Validate	Test	Train	Validate	Test
ANN	84.09%	82.62%	82.73%	84.28%	82.97%	83.02%
GCNN	77.09%	76.49%	76.59%	88.61%	88.64%	88.14%
M-specific ANN	87.64%	84.99%	84.86%	88.13%	86.19%	85.86%
M-Specific GCNN	91.16%	89.13%	89.23%	91.48%	90.04%	90.25%

**Table 5:** Confusion matrix for the M-specific GCNN predictions. Rows show the N, M, and S groups, while columns show the number of cells predicted for each group.

		Predicted Class		
		Normal	Mild	Severe
**True class**	Normal	145	12	44
	Mild	22	64	66
	Severe	15	21	1231

**Table 6:** Percentage of cells from normal, mild, and severe groups in the clusters assigned as macrophage subtypes.

Cluster/Subtype	Number of Cells	Percentage of Normal Cells	Percentage of Mild Cells	Percentage of Severe Cells
0/M1	6572	3.33%	3.45%	93.20%
5/M2	3544	29.00%	11.40%	59.50%
7/Macrophage	3241	18.30%	13.19%	68.40%
15/M1	1436	5.90%	28.03%	66.05%
18/M1	1218	13.90%	7.95%	78.90%
27/M1	255	0%	2.76%	97.20%

**Table 7:** Up-regulated DEGs in clusters identified as M1 macrophages.

Cluster	Total DEG *	Number of Unique DEG Genes **	Top 5 Up-Regulated (FC)
0	90	90	S100A8 (15.1), S100A9 (14.3), CCL2 (9.3), CXCL10 (8.3), IL1RN (8.1)
15	29	15	CST7 (3.5), RPS27(3.1), RPS19(2.6), ALOX5AP(2.5), XCL2(2.5)
18	40	29	IL32 (6.9), CD3E (3.7), CD2 (3.6), CORO1A (3.5), CD3D (3.3)
27	23	2	ZNF683 (2.0), BGLAP (2.1)

* Differentially expressed genes of one cluster compared to the rest of the clusters identified as M1 macrophages. ** Differentially expressed genes found only in one cluster.

**Table 8:** Down-regulated DEG in clusters identified as M1 macrophages.

Cluster	Total DEG *	Number of Unique DEG Genes **	Top 5 Down-Regulated (FC)
0	52	51	GNLY (0.03), GZMB (0.05), CCL5 (0.09), NKG7 (0.10), IL32 (0.11)
15	72	9	MT2A(0.4), ACTB (0.44), CSTB (0.44), S100A4 (0.46), HLA-DRA (0.47)
18	73	27	FOS (0.30), SRGN (0.36), NEAT1 (0.39), CCL4 (0.39), TNFSF10 (0.42)
27	66	0	N/A

* Differentially expressed genes of one cluster compared to the rest of the clusters identified as M1 macrophages. ** Differentially expressed genes found only in one cluster.

## Data Availability

The source code and data can be downloaded from https://github.com/Karladanielap/GeneSignaturesCOVID19 accessed on 10 November 2022.

## Supplementary Materials

The following supporting information can be downloaded at: https://www.mdpi.com/article/10.3390/genes13122264/s1, Figure S1: Quality control; Figure S2: UMAP of cells by patient; Table S1: Gene markers for cell type identification; Table S2: Up-regulated differentially expressed genes of M1 in Cluster 0 compared to M1 in clusters 15, 18, and27; Table S3: Gene ontology terms of up regulated genes of M1 cluster 0 (Genes on Table S2); Table S4: Down-regulated differentially expressed genes of M1 in Cluster 0 compared to M1 in clusters 15, 18, and 27; Table S5: Gene ontology terms of down regulated genes of M1 cluster 0 (Genes on Table S4).
