# Supplementary material for: Characterizing Macrophages Diversity in COVID-19 Patients Using Deep Learning
Source: Genes (Basel). 2022 Dec 1;13(12):2264. doi: 10.3390/genes13122264 (PMC9777824; doi:10.3390/genes13122264)
Supplement: Supplementary file 1 [file genes-13-02264-s001.zip › genes-1947304-supplementary.pdf]

Table S1. Gene markers for cell type identification

Cell type	Marker
Anterior foregut endoderm cell	CXCR4, EPCAM, SOX2
B cell	BANK1, BCL11A, BLK, CD19, MS4A1
Basal cell	ADH7, AQP3, CAV1, CCND2, CLCA2, CLCA4, CLDN1, COL17A1, CSTA, DSG3, FABP5, FGFBP1, GPNMB, GPX2, HSPB1, IGFBP6, IL1RN, JUP, KRT13, KRT14, KRT15, KRT16, KRT17, KRT4, KRT5, KRT6A, KRT6B, LAD1, LGALS7, LGALS7B, LY6D, MT1X, NRARP, PLAT, PTTG1, S100A10, S100A14, S100A16, S100A2, S100A8, SDC1, SERPINB13, SERPINB2, SERPINB5, SFN, SPINK5, SPRR1A, SPRR1B, THBD, TIMP1
CD4+ T cell	CD3D, CD3E, CD3G, IL7R, TRAC
CD8+ T cell	CD3D, CD3E, CD3G, CD8A, CD8B, FYN, GZMA, TRAC
Ciliated cell	AGR3, AKAP14, ALDH3A1, ANKRD37, ANKRD66, APOBEC4, ATF3, BASP1, BBOF1, C11orf97, C12orf75, C20orf85, C5orf49, C9orf24, CABCOCO1, CAPS, CAPSL, CCDC170, CCDC33, CCDC65, CCDC74B, CCDC81, CCL15, CDHR3, CETN2, CFAP126, CFAP299, CFAP52, CFAP53, CFAP73, CFAP77, CKB, CRIP1, CST6, CTXN1, CYP4B1, DAW1, DMKN, DNAAF3, DNAH12, DNAH7, DRC1, DYNLRB2, EFCAB10, ENKUR, ERICH3, EZR, FAM166B, FAM183A, FAM216B, FAM229B, FAM81B, FXYD3, GPR162, HBB, HSP90AA1, HYDIN, IFI27, IFT57, IGFBP7, KIF9, LRRC10B, LRRC23, LRRC46, LRRIQ1, METTL27, MIA, MORN2, MORN5, NME5, NQO1, PACRG, PIFO, PLAC8, PPIL6, PRDX5, PSCA, RBKS, RIIAD1, ROPN1L, RP1, RRAD, RSPH1, RSPH4A, SAA2, SAA2-SAA4, SAXO2, SCGB2A1, SMIM22, SNTN, SPA17, SPACA9, SPAG6, STMND1, STOML3, TEKT1, TMEM190, TMEM231, TPPP3, TSPAN1, TSPAN19, TUBA1A, TUBA4B, TUBB4B, VIM, VWA3B, WDR38, WDR54, ZDHHC1
Dendritic cell	CCR6, CCL20, CD1A, CD83, CLEC4C, THBD
Epithelial progenitor cell	CEACAM6
Fibroblast	CD36, FN1, VIM
M1 macrophage	CXCL10
M2 macrophage	CCL18, CD163
Macrophage	C1QA, CCL18, CCL2, CD14, CD163, CD83, CTSB, CXCL9, ICAM1, IL1RN, MRC1, MS4A6A, MSR1, TFRC
Monocyte	CD14, CD36, MRC1
Plasma cell	CD19, CD27, CD38, CD79A, IGHA1, IGHG1, IGHG4, IGHM, IGKC, JCHAIN, MZB1, SDC1, SLAMF7, XBP1
Secretory cell	ALDH1A3, ALDH1A1, BPIFB1, C3, CD74, CEACAM6, CP, CREB3L1, CXCL1, CXCL2, CXCL6, CXCL8, GABRP, HLA-DPA1, HLA-DPB1, HLA-DQA1, HLA-DQA2, HLA-DQB1, HLA-DRA, HLA-DRB1, HLA-DRB5, IFITM1, IFITM3, KRT18, LYPD2, MET, MSMB, MUC5B, NCCRP1, PDZK1IP1, PI3, PIGR, PLK2, PRSS23, RARRES1, SCGB1A1, SCGB3A1, SLC34A2, SLC6A14, SLPI, STEAP4, UBD, VMO1, XBP1, ZG16B
T cell	CD3D, CD3E, CD3G, CD8A
Type I pneumocyte	AQP5, CAV1, CD55, EMP2, HOPX, LMO7, PDPN, RTKN2, S100A14, S100A6
Type II pneumocyte	LAMP3, NAPSA, PGC, SFTPA1, SFTPA2, SFTPC, SLC34A2
mDC	CD1C

Table S2. Up-regulated differentially expressed genes of M1 in Cluster 0 compared to M1 in clusters 15, 18, and 27.

Gene	avg_log2FC	pct.1	pct.2	p_val_adj	FC
S100A8	3.91408514	0.997	0.659	1.10E-300	15.07499
S100A9	3.84128293	0.997	0.68	1.10E-300	14.3331413
CCL2	3.22920882	0.991	0.78	1.10E-300	9.3775355
CXCL10	3.05277233	0.976	0.71	1.10E-300	8.29804988
IL1RN	3.01170305	0.994	0.677	1.10E-300	8.0651594
APOBEC3A	3.00875985	0.985	0.579	1.10E-300	8.04872271
CCL8	2.72228492	0.842	0.653	1.10E-300	6.59917154
S100A12	2.42080534	0.892	0.574	1.10E-300	5.35469847
BCL2A1	2.39656459	0.994	0.64	1.10E-300	5.26547833
CCL3L1	2.29993903	0.927	0.701	1.10E-300	4.92436953
CCL7	2.28379316	0.839	0.551	1.10E-300	4.86956588
CTSL	2.26067825	0.996	0.716	1.10E-300	4.79216721
SAT1	2.24436293	1	0.955	1.10E-300	4.73827827
C15orf48	2.23965554	0.948	0.655	1.10E-300	4.72284288
TIMP1	2.22126941	0.962	0.64	1.10E-300	4.66303549
FCN1	2.20004179	0.968	0.544	1.10E-300	4.5949265
GLUL	2.19480796	0.995	0.714	1.10E-300	4.57828718
LYZ	2.18038283	0.972	0.578	1.10E-300	4.53273819
CTSB	2.15890226	0.998	0.812	1.10E-300	4.46574929
MAFB	2.14374373	0.955	0.556	1.10E-300	4.41907291
TYMP	2.13641564	1	0.889	1.10E-300	4.39668339
FTH1	2.08413573	1	1	1.10E-300	4.24021003
CXCL11	2.07603785	0.838	0.516	1.10E-300	4.21647632
SOD2	2.01597418	0.978	0.711	1.10E-300	4.04453595
NCF1	2.01178917	0.963	0.665	1.10E-300	4.03282045
IDO1	2.00313336	0.911	0.613	1.10E-300	4.00869696
FTL	1.99677537	1	0.997	1.10E-300	3.99106942
IFIT2	1.98908774	0.988	0.781	1.10E-300	3.96985892
RSAD2	1.98578773	0.994	0.853	1.10E-300	3.96078869
SGK1	1.98233789	0.951	0.586	1.10E-300	3.95132876
PLEK	1.97058698	0.983	0.651	1.10E-300	3.91927548
CST3	1.85562764	0.994	0.68	1.10E-300	3.61909164
IFITM3	1.8531196	1	0.963	1.10E-300	3.61280552
NAMPT	1.82646248	0.982	0.706	1.10E-300	3.54666355
PLAUR	1.75156248	0.952	0.579	1.10E-300	3.36723049
DUSP6	1.74805872	0.925	0.556	1.10E-300	3.35906268
IFIT1	1.71695842	0.978	0.802	1.10E-300	3.28742601
DEFB1	1.7122345	0.836	0.442	1.10E-300	3.27667936
S100A6	1.70540356	1	0.979	1.10E-300	3.26120145
WARS	1.69013143	0.976	0.767	1.10E-300	3.22686098
TYROBP	1.66488032	1	0.846	1.10E-300	3.17087349

IFIT3	1.64195451	0.997	0.881	1.10E-300	3.12088351
IFI30	1.6134169	0.954	0.574	1.10E-300	3.05975661
VAMP5	1.6122559	0.986	0.814	1.10E-300	3.05729529
NINJ1	1.60489932	0.931	0.63	1.10E-300	3.04174522
ISG15	1.58979949	1	0.995	1.10E-300	3.01007511
CCL3	1.57183572	0.971	0.81	1.10E-300	2.97282743
GBP1	1.56265881	0.997	0.889	1.10E-300	2.95397743
CD14	1.51317098	0.842	0.421	1.10E-300	2.85436728

Table S3. Gene ontology terms of up regulated genes of M1 cluster 0 (Genes on Table S2).

GO Term	Genes	P-value
GO:0006935-Chemotaxis	CCL2, CCL3L1, CCL3, CCL7, CCL8, CXCL10, CXCL11, CXCL8, S100A8, S100A9, FPR1, TYMP	4.30E-12
GO:0006954-Inflammatory response	CCL2, CCL3, CCL7, CCL8, CXCL10, CXCL11, CXCL8, CD14, CD163, S100A12, S100A8, S100A9, GBP5, NINJ1	1.10E-11
GO:0002376-Immunity	CD14, CD300E, IFI30, ISG15, S100A12, S100A8, S100A9, APOBEC3A, CLU, FCN1, GBP1, GBP5, IDO1, IFI27, IFIT1, IFIT2, IFIT3, IFITM3, IL4I1, LILRA5, HLA-DRA, RSAD2, TYROBP	4.70E-10
GO:0045087-Innate immunity	CD14, ISG15, S100A12, S100A8, S100A9, APOBEC3A, CLU, FCN1, IFI27, IFIT1, IFIT2, IFIT3, IFITM3, LILRA5, RSAD2	1.10E-08
GO:0051607-Antiviral defense	ISG15, APOBEC3A, GBP1, IFI27, IFIT1, IFIT2, IFIT3, IFITM3, RSAD2	5.40E-07
GO:0006915-Apoptosis	BCL2A1, S100A8, S100A9, TNFSF10, CLU, IFI27, IFIT2, SRGN, SGK1	7.90E-03

Table S4. Down-regulated differentially expressed genes of M1 in Cluster 0 compared to M1 in clusters 15, 18, and 27.

Gene	avg_log2FC	pct.1	pct.2	p_val_adj	FC
GNLY	-4.8889	0.408	0.778	1.10E-300	0.033752
GZMB	-4.11407	0.441	0.852	1.10E-300	0.057749
CCL5	-3.47572	0.412	0.891	1.10E-300	0.089889
NKG7	-3.28169	0.461	0.875	1.10E-300	0.102828
IL32	-3.1459	0.434	0.89	1.10E-300	0.112977
GZMA	-2.9777	0.403	0.89	1.10E-300	0.126947
CD7	-2.46053	0.38	0.884	1.10E-300	0.18168
STMN1	-2.32011	0.291	0.832	1.10E-300	0.200253
PRF1	-2.29382	0.276	0.829	1.10E-300	0.203935
HMGB2	-2.09781	0.379	0.811	1.10E-300	0.233613
CD2	-1.94554	0.419	0.88	1.10E-300	0.259618
CTSW	-1.86637	0.388	0.775	1.10E-300	0.274263
RPS27	-1.81042	0.996	1	1.10E-300	0.285109
RPS19	-1.7425	0.991	1	1.10E-300	0.298851
RAC2	-1.66309	0.653	0.959	1.10E-300	0.315762
RPL41	-1.64061	1	1	1.10E-300	0.32072
CST7	-1.57656	0.469	0.805	1.10E-300	0.335281
CORO1A	-1.5726	0.709	0.928	1.10E-300	0.336203
CD3E	-1.56684	0.439	0.755	1.10E-300	0.337546
KLRD1	-1.55706	0.275	0.702	1.10E-300	0.339843
LIMD2	-1.39869	0.393	0.862	1.10E-300	0.379274
LAG3	-1.3984	0.448	0.806	1.10E-300	0.37935
PTPRCAP	-1.37514	0.298	0.861	1.10E-300	0.385515
TYMS	-1.37205	0.282	0.779	1.10E-300	0.386343
KLRC1	-1.35196	0.396	0.666	1.10E-300	0.391759
RPL34	-1.34192	0.983	1	1.10E-300	0.394494
C12orf75	-1.29769	0.353	0.763	1.10E-300	0.406777
RPL13A	-1.26885	0.768	0.981	1.10E-300	0.41499
RPL21	-1.24814	0.918	0.997	1.10E-300	0.42099
CD3D	-1.23247	0.319	0.682	1.10E-300	0.425589
CD52	-1.21734	0.771	0.931	1.10E-300	0.430075
LTB	-1.21637	0.416	0.711	1.10E-300	0.430363
CD247	-1.21267	0.44	0.834	1.10E-300	0.43147
ALOX5AP	-1.20627	0.469	0.833	1.10E-300	0.433389
TMSB4X	-1.19248	0.999	1	1.10E-300	0.43755
ZFP36L2	-1.18298	0.397	0.857	1.10E-300	0.44044
XCL1	-1.18291	0.431	0.603	1.10E-300	0.440462
TRBC2	-1.17556	0.321	0.791	1.10E-300	0.442712
MT-ND4L	-1.14999	0.972	0.996	1.10E-300	0.450629

STK17A	-1.14328	0.529	0.872	1.10E-300	0.452728
IL2RB	-1.1269	0.407	0.79	1.10E-300	0.457899
UBE2C	-1.10553	0.409	0.74	1.10E-300	0.464731
HOPX	-1.10021	0.327	0.694	1.10E-300	0.466449
LCK	-1.09124	0.451	0.739	1.10E-300	0.469359
RGS1	-1.08452	0.208	0.765	1.10E-300	0.471549
GIMAP7	-1.07559	0.542	0.825	1.10E-300	0.474478
GZMH	-1.05476	0.311	0.646	1.10E-300	0.481379
TRDC	-1.05	0.283	0.69	1.10E-300	0.482969
MKI67	-1.03331	0.382	0.764	1.10E-300	0.488588
SH2D2A	-1.00928	0.393	0.785	1.10E-300	0.496793
CD96	-1.00509	0.457	0.779	1.10E-300	0.49824

Table S5. Gene ontology terms of down regulated genes of M1 cluster 0 (Genes on Table S4).

GO Term	Genes	P-value
GO:0019835-Cytolysis	GZMA, GZMB, GZMH, NKG7, PRF1	1.1E-7
GO:0002250-Adaptive Immune response	CD247, CD3D, CD3E, CD7, TRBC2, TRDC, KLRC1, KLRD1, LAG3	3.8E-5
GO:0002376-Immunity	CD14, CD300E, IFI30, ISG15, S100A12, S100A8, S100A9, APOBEC3A, CLU, FCN1, GBP1, GBP5, IDO1, IFI27, IFIT1, IFIT2, IFIT3, IFITM3, IL4I1, LILRA5, HLA-DRA, RSAD2, TYROBP	5.4E-4
GO:0005840-Ribosomal protein	RPL13A, RPL21, RPL34, RPL41, RPS19, RPS27	6.6E-3

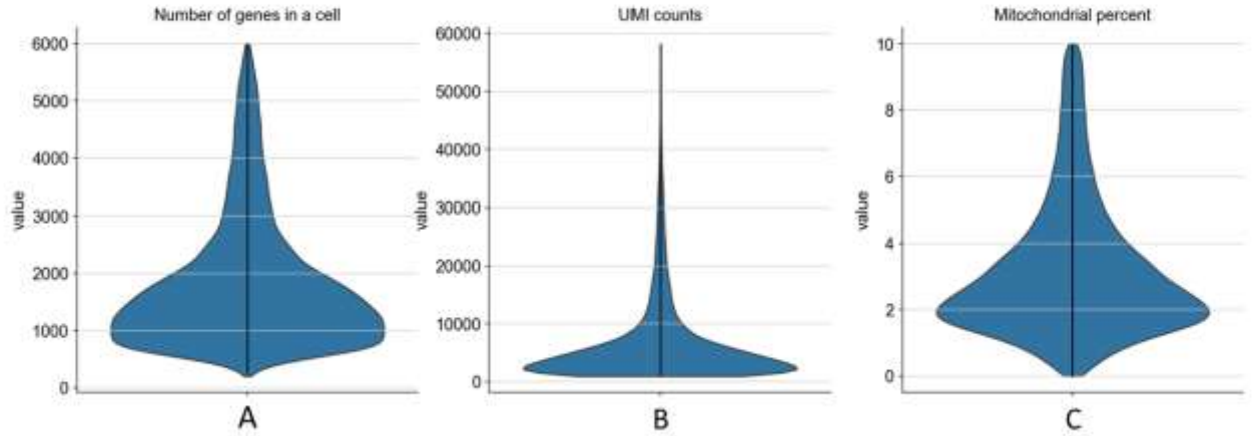

Figure S1. Quality control. Violin plot for 63,734 cells after applying three filters with three criteria: 1) the number of genes in a cell should stay between 200 and 6,000, UMI counts greater than or equal to 1,000, and percentage of mitochondrial less than or equal to 0.1. (A) Violin plot of the number of genes detected in cells after filtering; (B) Violin plot of the UMI counts in cells after filtering; (C) Violin plot of the percentage of mitochondrial in cells after filtering.

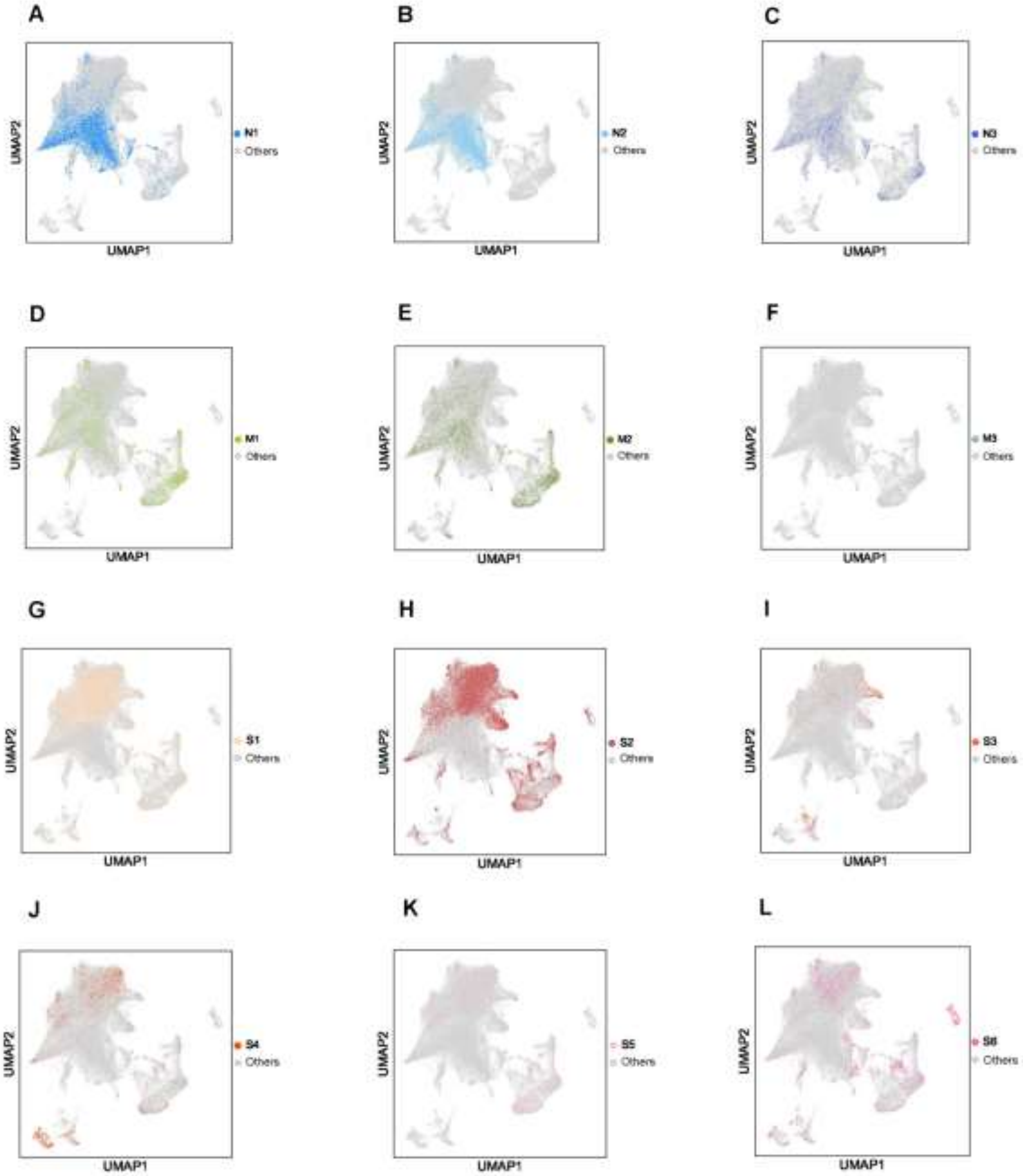

Figure S2. UMAP of cells by patient. A-C showing the UMAP of the cells belonging to Normal patients. D-F the UMAP of cells belonging to Mild patients. G-L The UMAP of cells belonging to Severe patients. Gray color shows the cells that belong to the rest of the patients.
